# On the Dropsy Which Follows Scarlatina

**Published:** 1845-09

**Authors:** 


*On the Dropsy which follows Scarlatina.*—Dr. Golding Bird gives the following summary of the facts recognized in connection with the development of dropsy after scarlatina:—

1. The anasarca does not appear during the existence of the rash

2. The sequelæ, which do not depend on local mischief about the throat, usually appear about the end of the first week after the recession of the rash, rarely before, and not often after this period.

3. The frequency of their occurrence is in the inverse ratio of the vividity of the rash.

4. The urine contains certain of the elements of the blood (albumen and red particles,) with a considerable number of large organic globules.

5. The blood contains some of the elements of urine, as proved by the existence of urea in it, as well as in the secretions derived from it.

6. Analogous effects, although looked for, have not been observed on the recession of other exanthema, as measles and small-pox; nor in cutaneous affections, in which free perspiration must be checked or greatly lessened, as in lepra, psoriasis, chronic eczema, &c.—*Ibid.*

